# Natural Densitals

**DOI:** 10.1021/acs.jpclett.4c03166

**Published:** 2025-01-10

**Authors:** Jerzy Cioslowski, Krzysztof Strasburger

**Affiliations:** † Institute of Physics, University of Szczecin, Wielkopolska 15, 70-451 Szczecin, Poland; ‡ Max-Planck-Institut für Physik komplexer Systeme, Nöthnitzer Straße 38, 01187 Dresden, Germany; § Faculty of Chemistry, Department of Physical and Quantum Chemistry, Wrocław University of Science and Technology, Wybrzeże Wyspiańskiego 27, 50-370 Wrocław, Poland

## Abstract

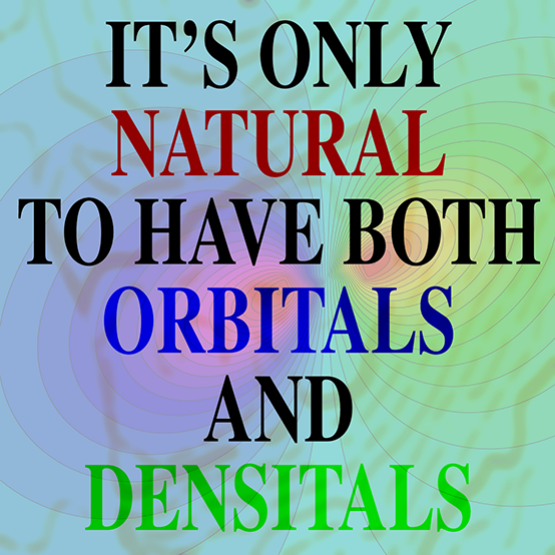

The concept of natural densitals (NDs) and their amplitudes is introduced. These quantities provide the spectral decomposition of the cumulant  of the two-electron density that, by definition, quantifies the extent of electron correlation. Consequently, they are ideally suited for a rigorous description of electron correlation effects in Coulombic systems. Spin-summed and spin-resolved versions of the NDs and their amplitudes are defined, and their properties are discussed in detail. Unlike the nonnegative-valued occupation numbers of the natural orbitals (NOs), these amplitudes exhibit diverse sign patterns that emerge within different regimes of electron correlation. The descriptive power of this property is vividly illustrated with the ground state of the H_2_ molecule, in which the subtle interplay of various types of electron correlation is captured in detail by a straightforward examination of the amplitudes of the NDs alone. Offering the most compact bilinear representations of  (a property analogous to that of the NOs with respect to the 1-matrix), the NDs open up entirely new vistas in the analysis of electronic structures of atoms and molecules.

Thanks to the interactions in Coulombic systems involving at most two particles, any electronic property of an atom, a molecule, or an extended system can be directly computed from the pertinent two-electron reduced density matrix (the 2-matrix)
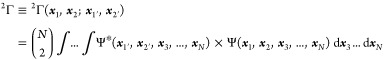
1defined in terms of the underlying *N*-electron wave function Ψ(***x***_1_, ***x***_2_, ***x***_3_, ..., ***x***_*N*_).^1,2^ In particular, the kinetic energy, the energy of interaction with the external potential (such as that due to nuclei), and the electron-electron repulsion energy, are given by explicit functionals of the one-electron reduced density matrix (the 1-matrix)
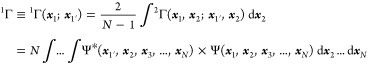
2its diagonal part, i.e., the one-electron density
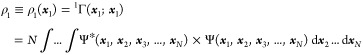
3and the diagonal part of ^2^Γ, i.e., the two-electron (a.k.a. pair) density
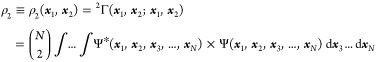
4respectively. However, despite ^1^Γ, ρ_1_, and ρ_2_ following trivially from ^2^Γ, the necessity of imposing extremely complicated *N*-representability constraints renders direct minimization of the electronic energy with respect to the 2-matrix computationally unfeasible.^3,4^

One infers from the interrelations 2−4 that the knowledge of the full 2-matrix is not a prerequisite for the computation of the nonrelativistic electronic energy as all of its components (as well as virtually all other electronic properties of practical importance) can be obtained from just two quantities, namely, ^1^Γ(***x***_1_; ***x***_1′_) and the cumulant of the two-electron density (note its sign convention that is commonly used in quantum chemistry but is opposite to that used by physicists)^5,6^
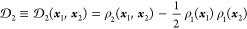
5

Both ^1^Γ(***x***_1_; ***x***_1′_) and  are given by sums involving products of Cartesian and spin components, i.e., ^1^Γ(***x***_1_; ***x***_1′_) =  +  as well as  =  +  +  +  +  + , from which the spin-summed quantities (with the different arguments)  =  +  and  =  +  +  +  follow. Since the cumulant  = , which originates from the *αβαβ* spin block of the 2-matrix, enters the products that do not survive the spin summation, it is excluded from further considerations in this Letter.

It should be noted that ^1^Γ(***x***_1_; ***x***_1′_) and  share the important property of additive separability (i.e.,  =  +  as well as  =  + , where the subscripted letters in square brackets refer to two noninteracting systems *A* and *B*) that ρ_2_(***x***_1_, ***x***_2_) lacks [as  =  +  +  + ]. As revealed by the inspection of eqs 2–5, ^1^Γ and  are related through the identity
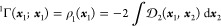
6and thus not fully independent. Moreover, in the case of spin-unpolarized systems, the recently discovered cusp condition^7^
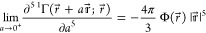
7links the spin-summed 1-matrix  with the on-top two-electron density
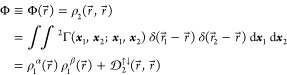
8where  is the spin-summed counterpart of ρ_2_(***x***_1_, ***x***_2_),  = , , and  =  +  = .

Thus far, the equal importance of the roles played by ^1^Γ and  in the manifestly size-consistent formulation of the electronic structure theory described above has not translated into equal attention paid in the literature to the elucidation of their properties. For example, the published approaches to turning these quantities (whose Cartesian components are not readily visualizable six-dimensional objects) into those amenable to facile interpretation differ significantly. One of such approaches involves spin summation of the diagonal parts of ^1^Γ and  that produces ^1^Γ(*r⃗*; *r⃗*) = ρ_1_(*r⃗*) = ρ_1_^α^(*r⃗*) + ρ_1_^β^(*r⃗*) and  = , respectively. The first of the resulting quantities, i.e., the spin-summed one-electron density , is the mainstay of interpretive quantum-chemical formalisms (such as the theory of atoms in molecules that provides both an unequivocal delineation of dominant interatomic interactions and a rigorous Cartesian-space partitioning of electronic properties into atomic contributions^8^). In stark contrast, analogous studies of the quantities related to  have been scarce. Due to the undesirable conflation of the topological features of  and , the choice of the so-called on-top ratio  as the object of these studies has been found to produce critical points in large numbers, obscuring their interpretation even for the simplest atoms and molecules.^9^ On the other hand, the alternative approach to the visualization of  that involves plotting the correlation factor  ^10^ or the exchange-correlation hole density  ^11^ vs  for a series of reference vectors  suffers from arbitrariness in the choice of the latter.

The aforedescribed contractions  and  are inevitably associated with some loss of information that, in the case of ^1^Γ, is entirely avoided upon the introduction of the spectral (or, more precisely, Schmidt) decomposition^12^
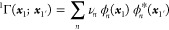
9where the functions {ϕ_*n*_} ≡ {ϕ_*n*_(***x***_1_)} (which form an orthonormal set) are the natural spinorbitals and {ν_*n*_} are the respective occupation numbers (ordered nonascendingly). The benefits of employing this decomposition, which features prominently in diverse formalisms of electronic structure theory,^13^ are manifold. First of all, being one-electron functions, the Cartesian components of the natural spinorbitals are readily visualized. Second, the availability of the occupation numbers allows facile construction of various measures of electron correlation.^14,15^ Third, thanks to the cusp condition 7, the *n* → *∞* asymptotics of the decay of ν_*n*_ with *n*(16−19) and those of the symmetry incidences of {ϕ_*n*_}^20^ can be rigorously derived, permitting the elucidation of the dependence of the errors in approximate electronic properties on the sizes of the basis sets employed in quantum-chemical calculations.^21−23^

Several spectral decompositions of the 2-matrix ^2^Γ(***x***_1_, ***x***_2_; ***x***_1′_, ***x***_2′_) have been reported in the literature, the most known among them involving two-electrons analogs of {ϕ_*n*_(***x***_1_)} called the natural (spin)geminals .^24^ The magnitude of the largest occupation number in the respective decomposition of the two-electron density cumulant  ^6^ has been found to measure the extent of the off-diagonal long-range order.^25^ A decomposition of ^2^Γ(***x***_1_, ***x***_2_; ***x***_1′_, ***x***_2′_) in terms of another set  of orthonormal functions has also been considered.^26^ However, quite surprisingly, an analogous treatment of  has not been investigated thus far.

As already argued in this Letter, ^1^Γ(***x***_1_; ***x***_1′_) and  are equally important in the size-consistent formulation of electron structure theory and thus should be treated on equal footing. This observation prompts the introduction of the spectral decomposition
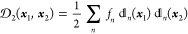
10that involves the entirely new concepts of natural densitals (NDs)  and their amplitudes  (ordered according to nonascending absolute values). In analogy to the spin-summed/spin-resolved versions of eq 9
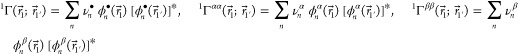
11that define three variants of the natural orbitals (NOs) and their occupation numbers, four distinct types of NDs and their amplitudes emerge from the decompositions
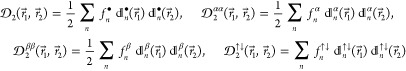
12(note that here and in the following, the quantities derived from the spin-summed ^1^Γ and  are denoted by superscripted bullets).

As both the NOs and NDs form orthonormal sets of one-electron functions, eqs 11 and 12 appear at the first glance to be very similar [although the absence of complex conjugations in the latter, as  in contrast to , should be noted]. However, inequalities analogous to those satisfied by the occupation numbers of the NOs that enter eq 11, namely , , and ,^24^ are currently unknown for the amplitudes of the NDs. On the other hand, the set of the sum rules for the NDs and their amplitudes is much richer than that pertinent to the occupation numbers of the NOs (i.e., , , and ,^24^ where *N*^α^ and *N*^β^ are the numbers of electrons with spins up and spins down, respectively). In particular, the contraction 6 implies
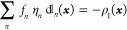
13where
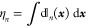
14is the 1-pseudonorm of the *n*th ND, and more specifically,
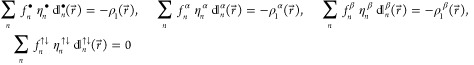
15where
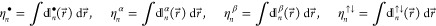
16In turn, the sum rules
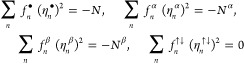
17derive directly from eq 15.

One infers from the above considerations that there are two types of the NDs. The active ones have nonvanishing (i.e., positive-valued by convention) 1-pseudonorms, whereas the 1-pseudonorms of the silent NDs equal zero. In general, only the NDs that belong to the same totally symmetric irreducible representation of the relevant point symmetry group (i.e., “have the same symmetry”) as  can be active. However, as easily deduced from the last component of the equation set 15, ; i.e., all the NDs  with nonvanishing amplitudes are silent and thus endowed with at least one nodal surface each. Nevertheless, these NDs enter the expression (which derives from eqs 8 and 12)
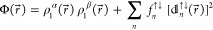
18from which the sum rule
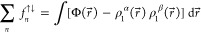
19readily follows. Another set of sum rules stems from the vanishing of the same-spin contributions to  at , which gives rise to
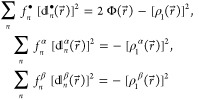
20upon combining eqs 5, 8, and 12. These rules complement that given by the identity 18. By the same token, the identity 19 is complemented by the sum rules
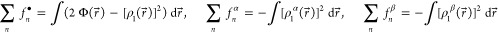
21that follow from eq 20.

The techniques of asymptotic estimation previously developed for the NOs^16^ are directly applicable to the NDs. Thus, as the cusp condition^27^
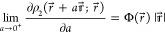
22for  carries over to , the leading terms  and  in the respective large-*n* asymptotics of  and  turn out to satisfy the zero-energy Schrödinger equation
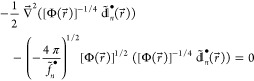
23from which various identities, such as
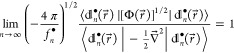
24
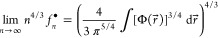
25and
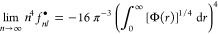
26follow. In eq 26,  are the amplitudes of the NDs  [where  is the spherical harmonic] with a given angular momentum  that are relevant to electronic states with spherically symmetric . As expected from the comparison of the cusp conditions 7 and 22, eqs 23–26 are obtained from their counterparts derived for the NOs^16^ by replacing ν_*n*_, , , , and  with , , , , and , respectively (while keeping in mind that both  and  are nonpositive-valued). Because of these analogies, the NDs with large *n* (or  are expected to have the nodal structures and the overall shapes of the isosurfaces (i.e., the level sets) similar to those of their weakly occupied NO counterparts, the dissimilarities being exhibited mainly in the respective spatial extents.

Highly accurate numerical data computed for some two-electron systems in singlet ground states vividly illustrate the aforediscussed properties of the NDs. The electronic wave functions of such systems are given by products of spatial and spin components, i.e.,  − , giving rise to particularly simple expressions for the on-top density, the spin-summed/spin-resolved one-electron density, and the spin-summed/spin-resolved cumulant of the two-electron density that read ,  = ,  =  = , and  =  =  =  − , respectively. Consequently, reflecting the absence of the same-spin electron pairs, the sets  ≡  and  ≡  each comprise just one ND (with a negative-valued amplitude) that is proportional to . Quite importantly, the cusp condition 22 also holds for , implying that eqs 23–26 remain valid upon the substitutions  → ,  → ,  → , and  → .

These predictions are borne out by the NDs computed for the ground state of the helium atom.^28^ The amplitudes  of the NDs with  are all negative and, as revealed by inspection of Figure 1, already approach the asymptotics (compare eq 26)
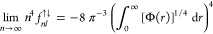
27for relatively small values of . Equally rapid convergence is observed (Figure 2) for the ratios  defined as (compare eq 24)
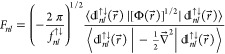
28Since eq 27 and the asymptotics  follow directly from the cusp condition 22 that stems from the short-range electron-electron repulsion, the close conformity of the computed values of  and  to their asymptotic estimates draws a clear picture of predominantly dynamical electron correlation. This conclusion is reinforced by the observed uniform negative-valuedness of the amplitudes whose deviation from such a sign pattern would indicate the presence of significant nondynamical correlation effects that go beyond the introduction of derivative discontinuities in electronic wave functions.

**Figure 1:**
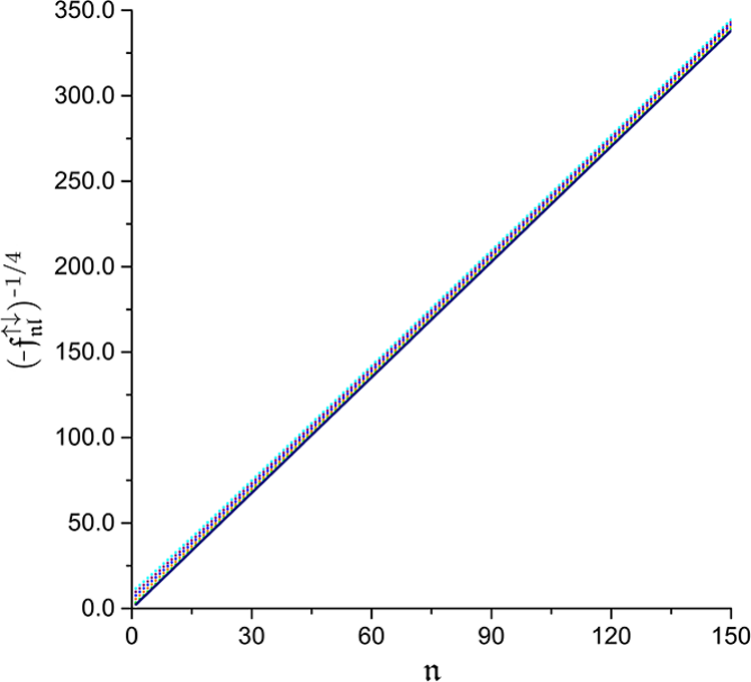
Verification of the asymptotics 27 for  (red),  (green),  (orange),  (blue),  (purple), and  (cyan). The dots correspond to the data computed for the ground state of the helium atom, whereas the dark blue line has the slope .

**Figure 2:**
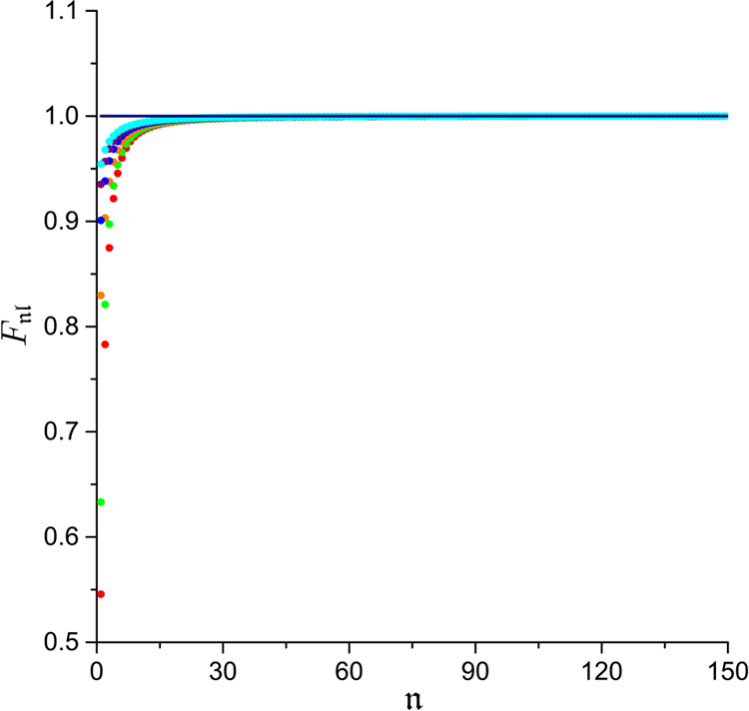
Verification of the asymptotics  for  (red),  (green),  (orange),  (blue),  (purple), and  (cyan). The dots correspond to the data computed for the ground state of the helium atom, whereas the dark blue line marks the limiting value of 1.

An entirely different situation is encountered with the amplitudes of the NDs pertaining to the ground state of the H_2_ molecule,^29^ whose evolution with the internuclear distance *R*_*HH*_ (Figure 3; note that the NDs are indexed in accordance with their ordering at small values of *R*_*HH*_) reveals the presence of three distinct regimes, each characterized by a different interplay between the dynamical and nondynamical (i.e., left-right) electron correlation. The transitions between these regimes are delineated by two landmarks in the amplitude of the 3σ_*g*_ ND as a function of *R*_*HH*_, namely, its zero at  and its maximum at . The first of these landmarks signals the breakdown of the simple picture of predominant short-range electron correlation that, per the aforementioned argument, is incompatible with the positive-valued amplitudes of the NDs, whereas the second one signifies the emergence of a prevailing mode of electron correlation that eventually becomes exclusive at the dissociation limit.

**Figure 3:**
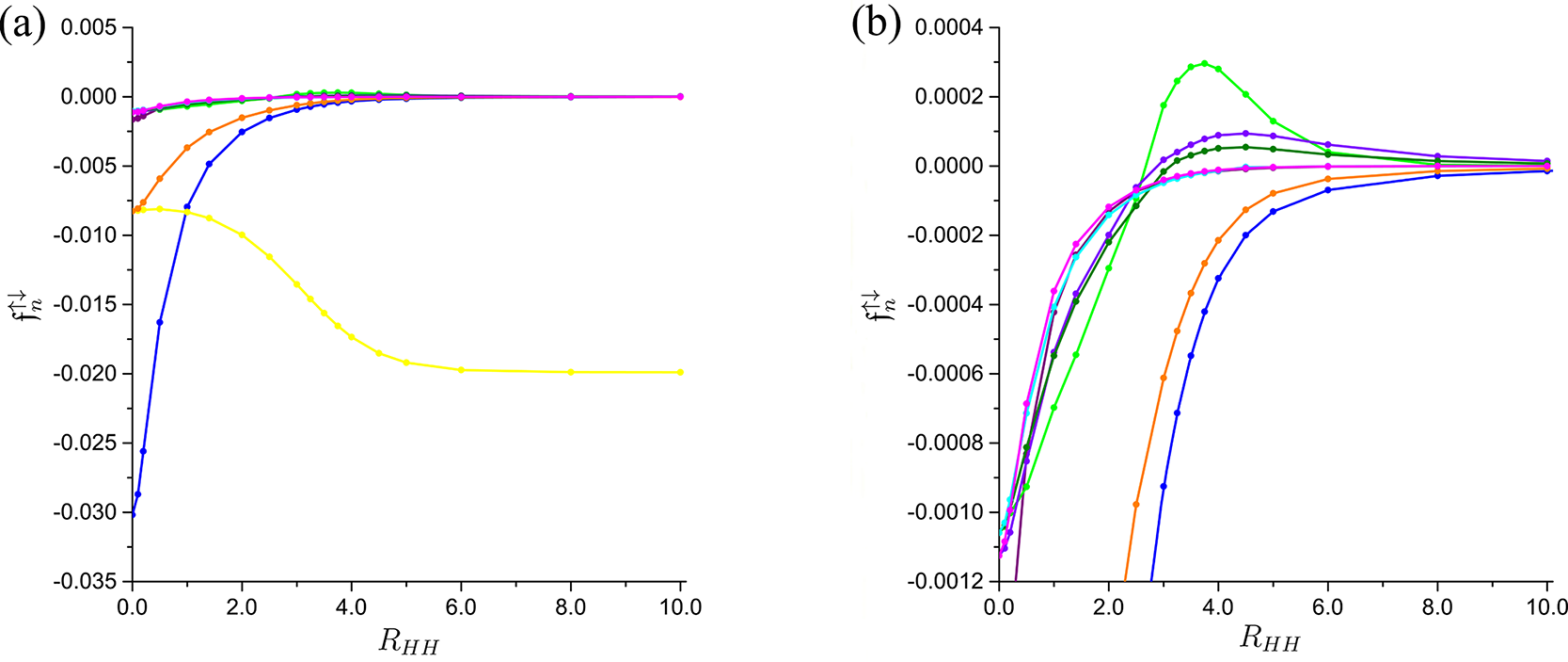
The evolution of the amplitudes  of the 1σ_*g*_ (blue), 1σ_*u*_ (yellow), 1π_*u*_ (orange), 2σ_*g*_ (purple), 3σ_*g*_ (green), 2σ_*u*_ (violet), 1π_*g*_ (olive), 1δ_*g*_ (cyan), and 2π_*u*_ (magenta) NDs pertaining to the ground state of the H_2_ molecule with the internuclear distance *R*_*HH*_: a) the global view of all the nine amplitudes with the greatest absolute values (at small *R*_*HH*_) and b) a detailed view of some of these amplitudes. The lines are provided for eye guidance only.

Within the regime of the predominance of the dynamical electron correlation, which extends from the united atom limit to , the amplitudes of all the NDs remain negative-valued. As , this sign pattern (whose persistence beyond the equilibrium internuclear distance of 1.4011 is well in line with that of the nodal patterns of the corresponding NOs^16^) implies each ND contributing to the lowering of the electron-electron repulsion energy. The *R*_*HH*_ → 0 asymptotic behavior of the computed amplitudes is consistent with the expected 1*s* ↔ 1σ_*g*_, 1*p* ↔ {1σ_*u*_, 1π_*u*_}, 2*s* ↔ 2σ_*g*_, 2*p* ↔ {2σ_*u*_, 2π_*u*_}, 1*d* ↔ {3σ_*g*_, 1π_*g*_, 1δ_*g*_}, ... correspondences between the NDs pertaining to the ground states of the helium atom and the H_2_ molecule.

Within the transitional regime that extends from  to , the increase of the positive-valued amplitude of the 3σ_*g*_ ND with *R*_*HH*_ is concomitant with the passing of the amplitudes of the NDs such as 2σ_*u*_ and 1π_*g*_ through their respective zeros (which occur at the internuclear distances significantly greater than ). A complex picture of the electron-electron repulsion being lowered by the self-interactions of some NDs and raised by those of the others ensues from the resulting changes in the sign pattern. It should be emphasized that this picture is in *qualitative* variance with that offered by the often-quoted^30^ oversimplistic CASSCF(2,2) model, in which two σ-type NOs give rise to a pair of NDs with amplitudes having opposite signs (i.e., being positive-valued for the σ_*g*_ ND and negative-valued for its σ_*u*_ counterpart) at *all* internuclear distances.

The regime of the predominance of the left-right electron correlation commences at  and persists all the way to the dissociation limit. Within this regime, the amplitude of the 3σ_*g*_ ND decreases continuously with *R*_*HH*_ while remaining positively valued. The NDs gradually settle into a well-pronounced pattern of pairs, such as 1σ_*g*_/2σ_*u*_ and 1π_*g*_/1π_*u*_, whose components have amplitudes of approximately equal (diminishing with *R*_*HH*_) magnitudes but opposite signs. This behavior, which is a fingerprint of dispersion interactions,^31^ is reminiscent of that of the respective NOs.^32^

Throughout the aforedescribed evolution of the sign patterns, the amplitude of the 1σ_*u*_ ND remains negatively valued. At the limit *R*_*HH*_ → *∞*, this amplitude does not decay to zero but tends to  =  ≈ , where  is the 1*s* orbital of the hydrogen atom. This dependence on *R*_*HH*_ is consistent with the 1σ_*u*_ ND describing the left-right nondynamic correlation that becomes dominant at the dissociation limit.

Several observations concerning the aforediscussed example involving the ground state of the H_2_ molecule are in order here. First of all, it should be emphasized that the subtle interplay of various types of electron correlation is captured in detail by a straightforward examination of the amplitudes of the NDs alone. Needless to say, a wealth of further information can be extracted from the properties of the NDs themselves.^33^ This is so because, offering the most compact bilinear representations of the cumulant of the two-electron density (a property analogous to that of the NOs with respect to the 1-matrix^12,34^), the NDs open up entirely new vistas in analysis and computation of electron correlation effects. For example, the decomposition
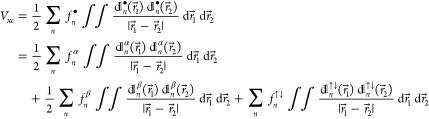
29permits examination of the exchange-correlation component *V*_*xc*_ of the electron-electron repulsion energy on the densital-by-densital basis. Together with its counterpart based upon the decomposition 18 for the on-top two-electron density, such an analysis opens an avenue to the identification of the densitals (either spin-summed or spin-resolved) contributing the most to different aspects of the electron exchange and correlation phenomena in a given Coulombic system and, in turn, delineation of the regions in the Cartesian space where these phenomena are most prominent. In this context, investigation of the linear combinations of the NDs that form the eigenfunctions of the , , , and  integral kernels is also of interest.

Second, it may appear at the first glance that an equally thorough analysis could be carried out with the natural amplitudes (whose squares equal the occupation numbers of the respective NOs) pertaining to the ground-state wave function of the H_2_ molecule. However, although these amplitudes exhibit sign patterns that indeed evolve with the internuclear distance,^31,35^ they are defined only for two-electron systems, which means that only the nonnegative-valued occupation numbers of the NO are available in the general case. With the loss of the information conveyed by the sign patterns, those numbers cannot yield the detailed description of electron correlation that is readily attained with the amplitudes of the NDs.

Third, being derived from the cumulant  that, by definition, quantifies the extent of electron correlation, the NDs and their amplitudes have the inherent property of being ideally suited for rigorous examinations of this phenomenon. In contrast, the influence of electron correlation on the 1-matrix is of an indirect nature, being reflected solely in the deviations of the nonidempotencies {ν_*n*_(1 – ν_*n*_)} from zero. Consequently, when employed in such examinations, various expressions involving these quantities^14,15^ can deliver much less information.

The concept of natural densitals affords a new addition to the instrumentarium of tools for the analysis of electron correlation in Coulombic systems. The possible directions of future research on this concept are manyfold, including the extension of the symmetry equiincidence principle^20^ to the densitals, the employment of these quantities in the detection of deficiencies in the description of electron correlation with various quantum-chemical formalisms, the construction of diverse indices such as those based upon the overlaps of the densitals with the one-electron density, and the investigation of the usefulness of the densitals derived from the  cumulant in the analysis of spin properties.

## References

[ref1] Throughout this Letter, the normalization *∫···∫* |Ψ|^2^ d***x***_1_ ... d***x***_*N*_ = 1, where *∫*d***x***_*k*_ stands for , is assumed. The atomic units and the standard notation ***x***_*k*_ ≡ (*r⃗*_*k*_, *σ*_*k*_) for the combined spatial and spin coordinates are used.

[ref2] Davidson E. R. Reduced Density Matrices in Quantum Chemistry; Academic Press: New York, 1976.

[ref3] Mazziotti D. A. Structure of Fermionic Density Matrices: Complete N-Representability Conditions. Phys. Rev. Lett. 2012, 108, 263002 10.1103/PhysRevLett.108.263002. 23004973

[ref4] Mazziotti D. A., Ed. Reduced-Density-Matrix Mechanics: With Application to Many-Electron Atoms and Molecules; Wiley: Hoboken, NJ, 2007.

[ref5] Ziesche P. Cumulant Expansions of Reduced Densities, Reduced Density Matrices, and Green’s Functions, in Many-Electron Densities and Reduced Density Matrices; Cioslowski J., Ed.; Kluwer Academic/Plenum Publishers: New York, 2000; pp 33–56.

[ref6] This *cumulant of the two-electron density* is distinct from the diagonal part of *the two-electron density cumulant* that enters the partitioning (note the sign convention) ρ_2_(***x***_1_, ***x***_2_) = – + .^5^

[ref7] Cioslowski J. Off-Diagonal Derivative Discontinuities in the Reduced Density Matrices of Electronic Systems. J. Chem. Phys. 2020, 153, 154108 10.1063/5.0023955. 33092376

[ref8] Bader R. F. W. Atoms in Molecules: A Quantum Theory; Clarendon Press: Oxford, 1994.

[ref9] Carlson R. K.; Truhlar D. G.; Gagliardi L. On-Top Ratio for Atoms and Molecules. J. Phys. Chem. A 2019, 123, 8294–8304. 10.1021/acs.jpca.9b04259. 31436419

[ref10] McWeeny R. Some Recent Advances in Density Matrix Theory. Rev. Mod. Phys. 1960, 32, 335–369. 10.1103/RevModPhys.32.335.

[ref11] Buijse M. A.; Baerends E. J. An Approximate Exchange-Correlation Hole Density as a Functional of the Natural Orbitals. Mol. Phys. 2002, 100, 401–421. 10.1080/00268970110070243.

[ref12] Löwdin P.-O. Quantum Theory of Many-Particle Systems. I. Physical Interpretations by Means of Density Matrices, Natural Spin-Orbitals, and Convergence Problems in the Method of Configurational Interaction. Phys. Rev. 1955, 97, 1474–1489. 10.1103/PhysRev.97.1474.

[ref13] See for example: Cioslowski J.; Strasburger K. Constraints upon Functionals of the 1-Matrix, Universal Properties of Natural Orbitals, and the Fallacy of the Collins ”Conjecture”. J. Phys. Chem. Lett. 2024, 15, 1328–1337. and the references cited therein 10.1021/acs.jpclett.3c03118. 38285733 PMC10860149

[ref14] See for example: Xu X.; Soriano-Agueda L.; López X.; Ramos-Cordoba E.; Matito E. All-Purpose Measure of Electron Correlation for Multireference Diagnostics. J. Chem. Theory Comput. 2024, 20, 721–727. 10.1021/acs.jctc.3c01073. 38157841 PMC10809408

[ref15] See for example: Ramos-Cordoba E.; Salvador P.; Matito E. Separation of Dynamic and Nondynamic Correlation. Phys. Chem. Chem. Phys. 2016, 18, 24015 10.1039/C6CP03072F. 27523386

[ref16] Cioslowski J.; Strasburger K. From Fredholm to Schrödinger via Eikonal: A New Formalism for Revealing Unknown Properties of Natural Orbitals. J. Chem. Theory Comput. 2021, 17, 6918–6933. 10.1021/acs.jctc.1c00709. 34672624

[ref17] Sobolev A. V. On the Spectrum of the One-Particle Density Matrix. Funct. Anal. its Appl. 2021, 55, 113–121. 10.1134/S0016266321020039.

[ref18] Sobolev A. V. Eigenvalue Estimates for the One-Particle Density Matrix. J. Spectr. Theory 2022, 12, 857–875. 10.4171/jst/407.

[ref19] Sobolev A. V. Eigenvalue Asymptotics for the One-Particle Density Matrix. Duke Math. J. 2022, 171, 3481–3513. 10.1215/00127094-2022-0032.

[ref20] Cioslowski J.; Strasburger K. Symmetry Equiincidence of Natural Orbitals. J. Phys. Chem. Lett. 2023, 14, 9296–9303. 10.1021/acs.jpclett.3c01738. 37815811

[ref21] Cioslowski J.; Strasburger K. A Universal Power Law Governing the Accuracy of Wave Function-Based Electronic Structure Calculations. J. Phys. Chem. Lett. 2022, 13, 8055–8061. 10.1021/acs.jpclett.2c01987. 35994623

[ref22] Cioslowski J.; Strasburger K. Angular-Momentum Extrapolations to the Complete Basis Set Limit: Why and When They Work. J. Chem. Theory Comput. 2021, 17, 3403–3413. 10.1021/acs.jctc.1c00202. 34003646

[ref23] Hill R. N. Rates of Convergence and Error Estimation Formulas for the Rayleigh-Ritz Variational Method. J. Chem. Phys. 1985, 83, 1173–1196. 10.1063/1.449481.

[ref24] Coleman A. J. Structure of Fermion Density Matrices. Rev. Mod. Phys. 1963, 35, 668–687. 10.1103/RevModPhys.35.668.

[ref25] Raeber A.; Mazziotti D. A. Large Eigenvalue of the Cumulant Part of the Two-Electron Reduced Density Matrix as a Measure of Off-Diagonal Long-Range Order. Phys. Rev. A 2015, 92, 052502 10.1103/PhysRevA.92.052502.

[ref26] Davidson E. R. N-Representability of the Electron Pair Density. Chem. Phys. Lett. 1995, 246, 209–213. 10.1016/0009-2614(95)01102-F.

[ref27] Tew D. P. Second Order Coalescence Conditions of Molecular Wave Functions. J. Chem. Phys. 2008, 129, 014104 10.1063/1.2945900. 18624467

[ref28] These NDs have been derived from a highly accurate 2354-term wavefunction with calculations involving bases comprising 500 one-electron functions; for details see: Cioslowski J.; Prątnicki F. Natural Amplitudes of the Ground State of the Helium Atom: Benchmark Calculations and Their Relevance to the Issue of Unoccupied Natural Orbitals in the H_2_ Molecule. J. Chem. Phys. 2019, 150, 074111 10.1063/1.5065791. 30795659

[ref29] These NDs have been derived from highly accurate wave function comprising between 1000 and 1192 terms with calculations involving bases composed of 258 one-electron functions.

[ref30] Carlson R. K.; Truhlar D. G.; Gagliardi L. On-Top Pair Density as a Measure of Left-Right Correlation in Bond Breaking. J. Phys. Chem. A 2017, 121, 5540–5547. 10.1021/acs.jpca.7b04259. 28653838

[ref31] Cioslowski J.; Schilling C.; Schilling R. 1-Matrix Functional for Long-Range Interaction Energy of Two Hydrogen Atoms. J. Chem. Phys. 2023, 158, 084106 10.1063/5.0139897. 36859076

[ref32] Cioslowski J.; Pernal K. Unoccupied Natural Orbitals in Two-Electron Coulombic Systems. Chem. Phys. Lett. 2006, 430, 188–190. 10.1016/j.cplett.2006.08.111.

[ref33] Due to the space limitations of this Letter, the presentation of this information is deferred to a future publication reporting on the analysis of electronic structures of several few-electron systems in terms of the NDs.

[ref34] The employment of the NOs and their occupation numbers assures the fastest possible rates of decay of the errors − , − , and − , defined in terms of the norm = , that are incurred upon truncations of the sums that enter the spectral representations 11.

[ref35] Cioslowski J.; Prątnicki F.; Strasburger K. Solitonic Natural Orbitals in Coulombic Systems. J. Chem. Phys. 2022, 156, 034108 10.1063/5.0075986. 35065571

